# Effects of hypoxia–reoxygenation on the bioenergetics and oxidative stress in the isolated mitochondria of the king scallop, *Pecten maximus*

**DOI:** 10.1242/jeb.249870

**Published:** 2025-05-12

**Authors:** Linda Lumor, Christian Bock, Felix Christopher Mark, Siriluck Ponsuksili, Inna Sokolova

**Affiliations:** ^1^ Institute for Farm Animal Biology (FBN), Institute of Genome Biology, 18196 Dummerstorf, Germany; ^2^ Department of Marine Biology, Institute for Biological Sciences, University of Rostock, 18059 Rostock, Germany; ^3^ Integrative Ecophysiology, Alfred Wegener Institute Helmholtz Centre for Polar and Marine Research, 27515 Bremerhaven, Germany; ^4^ Department of Maritime Systems, Interdisciplinary Faculty, University of Rostock, 18059 Rostock, Germany

**Keywords:** Marine bivalve, Mitochondrial phenotype, Succinate, Electron transport system, Reactive oxygen species

## Abstract

The king scallop (*Pecten maximus*) is a highly aerobic subtidal bivalve species vulnerable to fluctuations in oxygen availability. This study investigated the effects of short-term (15 min) and long-term (90 min) hypoxia–reoxygenation (H/R) stress on substrate-specific mitochondrial functions in the gill and digestive gland tissues of *P. maximus*, oxidizing substrates that engage mitochondrial Complex I (pyruvate, palmitate) and Complex II (succinate). Under normoxic conditions, scallop mitochondria preferentially oxidized pyruvate. H/R stress induced a significant decline in Complex I-driven ATP synthesis, increased proton leak and dysregulated fatty acid oxidation, indicating mitochondrial vulnerability to H/R stress. Following H/R, both tissues demonstrated a greater capacity for succinate oxidation than for Complex I substrates; however, long-term H/R exposure led to a reduction in respiratory coupling efficiency across all substrates. Notably, gill mitochondria exhibited more effective regulation of reactive oxygen species efflux and electron leak compared with digestive gland mitochondria under H/R stress. Despite these physiological changes, no evidence of oxidative damage was detected, suggesting the presence of a robust mitochondrial antioxidant defense. Collectively, these findings suggest that succinate oxidation plays an important role in stress recovery in *P. maximus*, providing insights into mitochondrial resilience and the management of oxidative stress during intermittent hypoxia.

## Teaser

**Summary:** A study on the impact of hypoxia–reoxygenation stress on mitochondrial function in *Pecten maximus* reveals substrate-specific mitochondrial responses and vulnerability during intermittent hypoxia.

## INTRODUCTION

Hypoxia, prevalent in aquatic environments because of factors such as organic matter overload, nutrient pollution and stratification, poses significant challenges in coastal areas impacted by anthropogenic activities (Avendaño-Alvarez et al., 2017; Conley et al., 2011). Permanent hypoxia, as observed in coastal dead zones, is incompatible with metazoan life; however, even shorter periods of hypoxia, resulting from tidal, diurnal or seasonal fluctuations in dissolved oxygen levels, present significant stress to marine organisms (Wallace et al., 2014). The ability to withstand oxygen fluctuations and hypoxia varies among organisms adapted to different habitats, with tolerance increasing from the subtidal zone, where infrequent and unpredictable hypoxic events occur, to the intertidal zone, home to organisms adapted to survive periodic hypoxia induced by low-tide emersion (Amorim et al., 2021, 2022; Thyrring and Peck, 2021).

Mitochondria emerge as critical targets of hypoxia and reoxygenation (H/R) stress because of their central role in aerobic energetics and signaling pathways. Hypoxia disrupts the mitochondrial electron transport system (ETS), slowing ATP synthesis and increasing reactive oxygen species (ROS) production (Solaini et al., 2010). In some hypoxia-tolerant species, such as intertidal bivalves and annelids, mitochondria can generate ATP anaerobically by using fumarate as an electron acceptor instead of oxygen. However, this pathway is less efficient in terms of ATP yield and leads to the accumulation of anaerobic byproducts such as succinate (Tielens et al., 2002; van Hellemond et al., 2003). Upon recovery from hypoxia, the surge in ROS levels can cause mitochondrial damage, leading to a collapse of the ETS, impaired oxidative phosphorylation (OXPHOS) and oxidative stress (Chouchani et al., 2014; Kalogeris et al., 2014). Despite these challenges, some intertidal and benthic organisms have evolved specialized adaptations in their metabolic machinery to survive such stress, exhibiting varying degrees of hypoxia tolerance (Adzigbli et al., 2022a; Sokolov et al., 2021; Sokolova et al., 2019). Notably, hypoxia-tolerant species such as intertidal bivalves and some intertidal fish species show high mitochondrial resilience to oxygen fluctuations and are able to sustain or even enhance mitochondrial functional traits, including OXPHOS and ETS activity, whereas hypoxia-intolerant species (e.g. terrestrial mammals) experience loss of ETS activity, mitochondrial membrane depolarization and oxidative stress (Adzigbli et al., 2022a,b; Gerber et al., 2019; Ouillon et al., 2021; Sokolov et al., 2019; Chouchani et al., 2014; Ivanina et al., 2016).

Mitochondrial flexibility and robustness are essential for stress adaptation, ensuring cellular survival and energy homeostasis (Cortassa et al., 2017; Galgani et al., 2008; Sugden et al., 2009). Mitochondria demonstrate metabolic flexibility by adjusting their metabolism based on substrate availability and the energetic and biosynthetic needs of the cell under stressful conditions, with shifts in metabolic intermediates influencing this process (Leverve and Fontaine, 2001; Quinlan et al., 2013). This plasticity is observed in mammals during nutrient scarcity, type 2 diabetes and hypoxia (Cadenas, 2018; Cortassa et al., 2017, 2019), and in invertebrates and rodents under thermal stress and oxygen limitation (Jørgensen et al., 2021; Oellermann et al., 2012; Roussel et al., 2023; Sokolov et al., 2021). However, the role of mitochondrial fuels in stress responses, particularly in non-model species such as marine invertebrates, is not well understood. This knowledge gap has sparked growing interest in exploring mitochondrial flexibility in organisms with varying degrees of hypoxia tolerance (Boutilier and St-Pierre, 2000; Galli and Richards, 2014; Sokolova et al., 2019; McDonald et al., 2018). Marine bivalves, in particular, serve as an excellent model group, with species demonstrating a broad range of hypoxia sensitivity and differing substrate utilization strategies in response to H/R stress (Diaz and Rosenberg, 2008; Gäde, 1983; Song et al., 2024).

Earlier studies have demonstrated that hypoxia-tolerant bivalves, such as oysters and blue mussels, exhibit a H/R resilient mitochondrial phenotype, characterized by the maintenance of mitochondrial proteome integrity, upregulation of protein quality control mechanisms and enhanced capacity to oxidize succinate during recovery (Adzigbli et al., 2024, 2022a; Falfushynska et al., 2020; Steffen et al., 2020, 2021, 2023). However, because of a limited number of studies on mitochondria in related hypoxia-sensitive species (Ivanina et al., 2016; Kraffe et al., 2008), it remains unclear whether these traits are specific to hypoxia tolerance or represent a broader mitochondrial phenotype shared across bivalves. To address this gap in our knowledge, we examined the mitochondrial responses to H/R in the hypoxia-sensitive scallop *Pecten maximus*, a subtidal bivalve species found in the Eastern Atlantic, the North Sea and the Mediterranean Sea (Jennings et al., 1999; Le Pennec et al., 2003). Scallops are unique among bivalves in their mobility, using swimming and jumping to evade predators (Artigaud et al., 2014; Tremblay and Guderley, 2014). During intense activity, scallops often experience functional anaerobiosis, as oxygen demand exceeds supply (Guderley and Tremblay, 2016). Their energy-intensive muscle activity and inability to fully close their shells make them highly reliant on oxygenated environments, rendering them particularly sensitive to H/R stress (Bock et al., 2019). Scallops' reliance on mitochondria during activity and recovery highlights the importance of understanding their mitochondrial responses to H/R stress.

In this study, we exposed isolated mitochondria from two tissues of *P. maximus* – the gill and digestive gland – to short-term (15 min hypoxia, 10 min reoxygenation) and long-term (90 min hypoxia, 10 min reoxygenation) H/R stress. Both tissues are metabolically important: the gills facilitate oxygen uptake and are highly sensitive to oxygen fluctuations, while the digestive gland is involved in digestion and energy storage (Kennedy et al., 1996). We hypothesized that the gill mitochondria would be more sensitive to H/R stress than those from the digestive gland, with long-term hypoxia leading to more significant mitochondrial damage and increased oxidative stress in the gill. Based on prior research suggesting succinate as a recovery fuel in hypoxia-tolerant bivalves (Adzigbli et al., 2022a; Sokolov et al., 2019, 2021), we speculated that *P. maximus* mitochondria would show compromised succinate oxidation capacity under H/R stress to a greater extent than observed in hypoxia-tolerant species such as mussels and oysters. To test these hypotheses, we measured resting and ADP-stimulated oxygen consumption rates, as well as ROS efflux, in isolated gill and digestive gland mitochondria of *P. maximus* under control conditions and two H/R regimes. We used pyruvate and palmitate as Complex I-linked substrates and succinate as a Complex II-linked substrate. Additionally, we assessed oxidative damage by measuring protein carbonyl content and lipid peroxidation (4-hydroxynonenal) in isolated gill mitochondria during both short-term and long-term H/R exposures. Our findings aim to elucidate mechanisms underlying hypoxia sensitivity and contribute to improved understanding of mitochondrial plasticity and resilience in marine bivalves with varying degrees of hypoxia tolerance (Sokolova et al., 2019).

## MATERIALS AND METHODS

### Chemicals

The chemicals used in this experiment were of analytical grade or higher and were purchased from Sigma Aldrich (Munich, Germany), Fisher Scientific (Schwerte, Germany) or Carl Roth (Karlsruhe, Germany).

### Animals

Adult king scallops *Pecten maximus* (Linnaeus 1758) were collected in the estuary of Vigo, Spain (42°14′46.6″N 8°44′18.5″W) at 10 m depth. The scallops were transported submerged in aerated water at 10°C by car to the Alfred Wegener Institute (AWI), Helmholtz Centre for Polar and Marine Research in Bremerhaven, Germany. On arrival, scallops were moved to the aquarium system in the AWI filled with North Sea water (10°C, salinity 32 practical salinity units, psu). The scallops were acclimated for a minimum of 4 weeks and fed 3 times weekly with a mixture of commercial algal blend (Nyos, PhytoMaxx) and a self-cultivated algal mixture including *Rhodomonas* sp., *Phaeodactylum tricornutum*, *Chaetocerus* sp. and *Isochrysis galbana* (minimum 3000 cells ml^−1^). No mortality was observed during the transport or maintenance in the aquaria.

### Mitochondrial isolation

Mitochondria were isolated from the scallops' gill and digestive gland tissues as described elsewhere (Adzigbli et al., 2022a). Tissues were homogenized in an isolation buffer [50 mmol l^−1^ NaCl, 300 mmol l^−1^ sucrose, 130 mmol l^−1^ KCl, 30 mmol l^−1^ Hepes, 8 mmol l^−1^ EGTA, 1% fatty acid-free bovine serum albumin (BSA), 50 μg l^−1^ aprotinin, 1 mmol l^−1^ phenylmethylsulfonyl fluoride (PMSF) at pH 7.5]. The mitochondria were isolated by differential centrifugation and suspended in ice-cold assay media (30 mmol l^−1^ Hepes, 550 mmol l^−1^ sucrose, 10 mmol l^−1^ glucose, 130 mmol l^−1^ KCl, 10 mmol l^−1^ NaCl, 1 mmol l^−1^ MgCl_2_, 10 mmol l^−1^ KH_2_PO_4_, pH 7.2, and 1% fatty acid free BSA). Both tissues were isolated from the same scallop and the experimental design of this study closely followed the approach described previously (Adzigbli et al., 2024).

### Mitochondrial oxygen consumption and ROS efflux rates

Mitochondrial oxygen consumption (*Ṁ*_O_2__) and ROS efflux were measured simultaneously in an Oxygraph 2k high-resolution respirometer (Oroboros, Innsbruck, Austria) integrated with DatLab 7.4 software at 15°C. A Clark-type electrode and fluorometer (Fluorescence-Sensor Green, 525 nm) incorporated into the Oxygraph 2k were used to measure *Ṁ*_O_2__ and ROS efflux of isolated mitochondria, respectively. The ROS efflux was measured as the release of H_2_O_2_ reflecting the net balance between mitochondrial H_2_O_2_ production and consumption (Jastroch et al., 2010). The detailed procedure for the calibration of the Clark-type electrode (*Ṁ*_O_2__) and fluorometer (ROS efflux) is outlined in Adzigbli et al. (2022a). To measure the mitochondrial functional indices and ROS efflux, we performed a substrate–uncoupler–inhibitor titration (SUIT) (Adzigbli et al., 2024). The mitochondrial substrate fuels used to measure the substrate-specific mitochondrial LEAK respiration were pyruvate (5 mmol l^−1^, combined with 2 mmol l^−1^ malate to spark respiration), palmitoyl-dl-carnitine (10 µmol l^−1^) and succinate (10 mmol l^−1^). Mitochondrial respiration and ROS efflux were measured in resting (LEAK) and phosphorylating (OXPHOS) states. LEAK respiration was determined as the baseline respiration of resting mitochondria, in the absence of ATP synthesis. Following ADP addition, the ADP-stimulated mitochondrial respiration rate was measured, which reflects the OXPHOS rate and serves as a proxy for ATP synthesis capacity.

### H/R stress exposures

Separate experiments were performed to assess the effects of short-term and long-term H/R stress on isolated mitochondria. The ADP-stimulated mitochondria were permitted to exhaust all oxygen in the chamber and upon reaching a near-anoxia state (∼0% O_2_), maintained for 15 min for short-term H/R stress and 90 min for long-term H/R stress. The oxygen tension was then raised to ∼50–80% of air saturation by opening the respirometry chamber. The mitochondria were allowed to recover for 10 min (reoxygenation), after which the chamber was closed. Post-hypoxic OXPHOS rate was measured, and oligomycin was added to inhibit mitochondrial *F*_1_F_O_-ATPase, allowing for the measurement of post-hypoxic LEAK respiration. Pilot studies with scallop mitochondria showed that under normoxic conditions, the difference in LEAK respiration rates between State II (prior to ADP addition) and State IV (after the addition of ADP and oligomycin) was below 5% (data not shown). The measurements from this experiment resulted in four respiration and ROS efflux values measured for each mitochondrial isolate: two values for LEAK respiration (pre- and post-H/R stress) and two for OXPHOS respiration (pre- and post-H/R stress). Parallel measurements under normoxic conditions during long-term exposures (∼90 min) confirmed that the observed effects were H/R induced rather than a result of mitochondrial viability loss over time. Mitochondrial functional parameters remained stable over 90 min under normoxia, indicating no decline in viability (data not shown).

The protein content of mitochondrial suspensions was measured using a Bio-Rad Bradford protein assay (Bio-Rad, Hercules, CA, USA) (Bradford, 1976) using BSA as a standard and corrected for the BSA content of the assay media. Mitochondrial respiration and ROS efflux rates were expressed as nmol O_2_ min^−1^ mg^−1^ protein and nmol H_2_O_2_ min^−1^ mg^−1^ protein, respectively. The respiratory control ratio (RCR) was calculated as the ratio of OXPHOS to LEAK respiration (Estabrook, 1967; Gnaiger, 2014). The fractional electron leak rate (FEL) was estimated as the ratio of H_2_O_2_ efflux to oxygen consumption.

### Measurement of oxidative lesions by ELISA

After measuring mitochondrial functional indices, aliquots of gill mitochondria (*n*=30; 5 per substrate and exposure group) were collected pre- and post-H/R stress and stored at −80°C for oxidative marker analysis.

Protein carbonyl (PC) levels, an indicator of oxidative protein damage, were measured following Winterbourn and Buss (1999) with modifications according to Adzigbli et al. (2024). Samples were lysed and diluted to 10 µg ml^−1^ protein with phosphate-buffered saline (PBS). Carbonyl standards were prepared by mixing oxidized and reduced BSA solutions to a final protein concentration of 10 μg ml^−1^. Aliquots of samples and standards were incubated overnight at 4°C on ELISA plates (Nunc Immuno Maxisorp, Thermo Scientific), then treated with 5 mmol l^−1^ 2,4-dinitrophenylhydrazine (DNPH) at room temperature in the dark. After sequential washes with PBS and ethanol/PBS (1:1 v:v), plates were blocked with BSA for 2 h, followed by incubation with primary anti-DNP antibody (1:1000, Sigma-Aldrich, MAB2223) and secondary antibody (1:10,000, 115-035-003, Jackson ImmunoResearch Laboratories Inc.), with washes between steps.

4-Hydroxynonenal (HNE), a lipid peroxidation marker, was measured using a modified protocol from Weber et al. (2013) and Borovic et al. (2006). Samples were lysed and diluted to 5 mg ml^−1^ protein with PBS. HNE standards were prepared by dissolving 4-HNE-dimethylacetal (Sigma-Aldrich) in 1 mmol l^−1^ HCl, with absorbance at 450 nm used to determine concentration. ELISA plates (Nunc Immuno Maxisorp, Thermo Scientific) were coated with 200 μl of 0.05 mol l^−1^ carbonate binding buffer (pH 9.6) and 20 μl of sample or standard, incubated overnight at 4°C, washed, and blocked with 5% fat-free dry milk. After additional washes, plates were incubated with primary anti-4-HNE antibody (1:1000, Abcam, ab48506) for 2 h at 37°C, followed by peroxidase treatment and secondary antibody (1:10,000, 115-035-003, Jackson ImmunoResearch Laboratories Inc.) incubation.

Detection of PC and HNE was performed using TMB/E (tetramethylbenzidine ultra-sensitive) horseradish peroxidase (HRP) substrate, and absorbance was measured at 450 nm after stopping the reaction with 2 mol l^−1^ sulfuric acid. PC and HNE levels were expressed as nmol mg^−1^ protein.

### Statistics

The data on mitochondrial parameters including *Ṁ*_O_2__ , ROS efflux, FEL, RCR and oxidative stress markers were tested for normality and screened for outliers. A two-way repeated measures ANOVA was used to examine the effects of the main factors (tissue and oxidized substrate, or H/R exposure and oxidized substrate) and their interactions on the studied mitochondrial parameters. H/R exposures were treated as a within-subject factor, while respiratory substrate was considered a between-subjects factor. As mitochondrial activity measurements under different oxygen exposures (control and H/R) were performed on the same mitochondrial isolate, H/R exposure was also treated as a within-subject factor. To assess tissue-specific substrate utilization, Tukey's honest significant differences (HSD) tests were used for comparisons of the group means. Paired Student's *t*-tests were applied to investigate H/R-induced effects on *Ṁ*_O_2__ and ROS efflux, as these values were measured pre- and post-H/R stress in the same mitochondrial isolate. Statistical analyses were performed using GraphPad Prism v.7.02 (GraphPad Software Inc., La Jolla, CA, USA) and IBM^®^ SPSS^®^ Statistics v.22.0.0.0 (IBM Corp., Armonk, NY, USA). A significance level of *P*<0.05 was used. For *Ṁ*_O_2__ and ROS efflux data, the sample size (*N*) was 8 for all groups, except where *N* was reduced because of the exclusion of 1–2 statistically significant outliers (*P*<0.05). For oxidative stress markers, the *N* was 5 for all measurements. Results were described according to evidence-based thresholds (Muff et al., 2022): *P*=0.049–0.011 (moderate evidence), *P*=0.01–0.001 (strong evidence) and *P*<0.001 (very strong evidence).

## RESULTS

### Tissue-specific mitochondrial *Ṁ*_O_2__ and ROS efflux in normoxia

A repeated measures two-way ANOVA revealed moderate to very strong evidence for the effects of oxidized substrates on all studied mitochondrial traits (Table S1). Additionally, tissue type (gill versus digestive gland) showed very strong evidence of an effect on ROS efflux rates and FEL. The interactions between tissue type and substrate showed no significant effects overall, with the exception of OXPHOS respiration rate (Table S1).

**Fig. 1.**
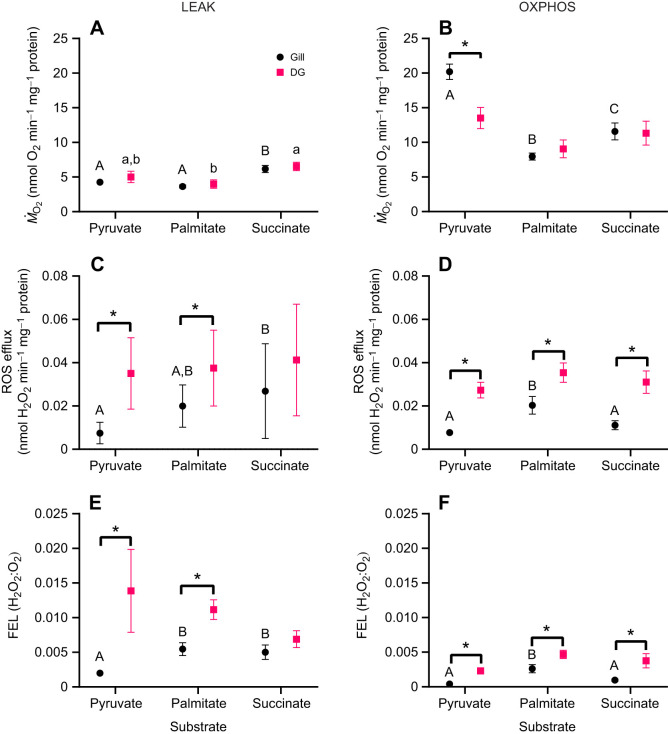
**Oxygen consumption rate (*Ṁ*_O_2__), reactive oxygen species (ROS) efflux and fractional electron leak (FEL) of *Pecten maximus* gill and digestive gland (DG) mitochondria.** *Ṁ*_O_2__ (A,B), ROS efflux (C,D) and FEL (H_2_O_2_:O_2_ ratio; E,F) in resting (LEAK; left) and active (OXPHOS; right) mitochondria respiring on Complex I substrates pyruvate and palmitate and Complex II substrate succinate. Significant effects of hypoxia exposure (between control and hypoxia–reoxygenation, H/R) on a particular mitochondrial trait are represented by asterisks (*P<0.05). *N*=16 for all substrates except in the gill mitochondria oxidizing pyruvate and palmitate (*N*=7 for each). Different uppercase and lowercase letters indicate a significant difference between oxidized substrates within each studied tissue (the gill and DG, respectively).

In general, the LEAK respiration rate was highest when succinate was used as a substrate, while the OXPHOS respiration rate was highest with pyruvate in mitochondria from both tissues (Fig. 1A,B). In the gill mitochondria, the ROS efflux rate in the LEAK state followed the order: pyruvate<palmitate<succinate (Fig. 1C). In the OXPHOS state, ROS efflux in the gills was highest during palmitate oxidation compared with pyruvate or succinate oxidation (Fig. 1D). A similar, though statistically non-significant, trend in substrate-dependent ROS efflux was observed in the digestive gland mitochondria (Fig. 1C,D). Interestingly, substrate-specific ROS efflux rates and FEL were higher in the digestive gland mitochondria compared with those in the gill, despite the digestive gland showing similar or lower mitochondrial respiration rates than the gills (cf. Fig. 1C–F and A,B).

### Effect of H/R stress on scallop mitochondria: LEAK state

In isolated gill mitochondria, LEAK state respiration and ROS efflux showed moderate to very strong evidence of interactive effects between H/R exposure and oxidized substrate, indicating that the response to H/R was substrate dependent (Tables S2 and S3). In the digestive gland mitochondria, only short-term H/R exposure, and not long-term H/R, showed evidence of a significant interaction with oxidized substrate on mitochondrial respiration, though no such interaction was observed for ROS efflux (Tables S2 and S3).

**Fig. 2.**
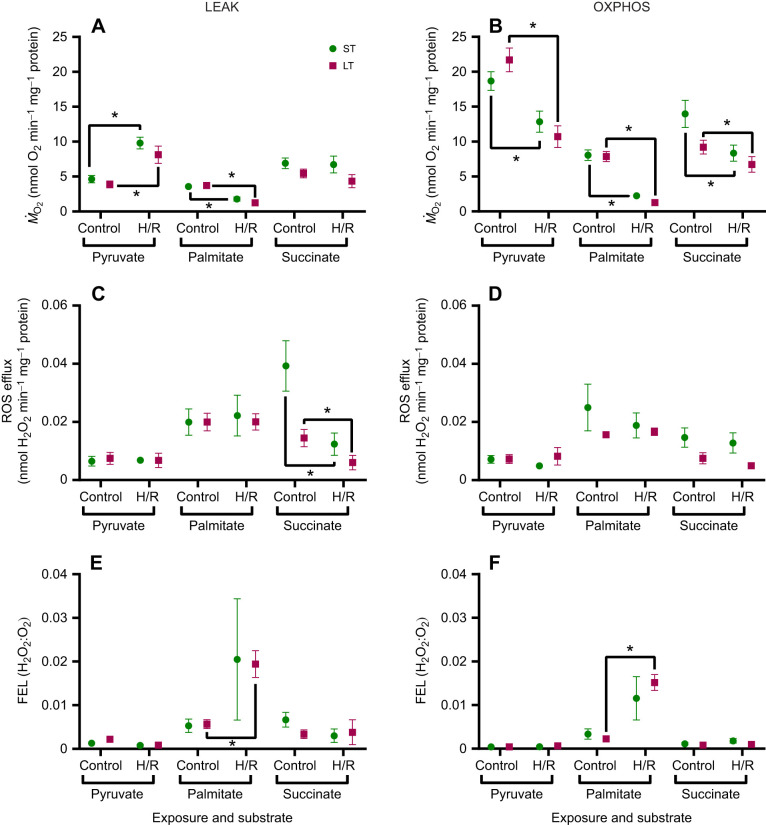
**Effects of short-term (ST) and long-term (LT) hypoxia–reoxygenation (H/R) on LEAK and OXPHOS respiration of *P. maximus* gill mitochondria.** *Ṁ*_O_2__ (A,B), ROS efflux (C,D) and FEL (E,F) in resting (LEAK; left) and active (OXPHOS; right) mitochondria oxidizing pyruvate, palmitate and succinate. Significant effects of hypoxia exposure (between control and H/R) on a particular mitochondrial trait are represented by asterisks (*P<0.05). *N*=8 for all substrates except palmitate (*N*=7 for palmitate-driven ROS and FEL in long-term hypoxia).

Both short- and long-term hypoxia increased LEAK state oxygen consumption in gill mitochondria oxidizing pyruvate, without a simultaneous increase in ROS efflux or FEL (Fig. 2A,C,E). Short- and long-term hypoxia suppressed the palmitate-driven LEAK respiration. However, LEAK state ROS efflux did not change, while FEL increased after H/R in mitochondria oxidizing palmitate (Fig. 2C,E). There was no evidence for a H/R effect on the succinate-driven LEAK *Ṁ*_O_2_ _of the gill mitochondria (Fig. 2A). The ROS efflux in the LEAK state decreased after H/R, and FEL did not change in response to H/R in the LEAK state gill mitochondria respiring on succinate (Fig. 2C,E).

**Fig. 3.**
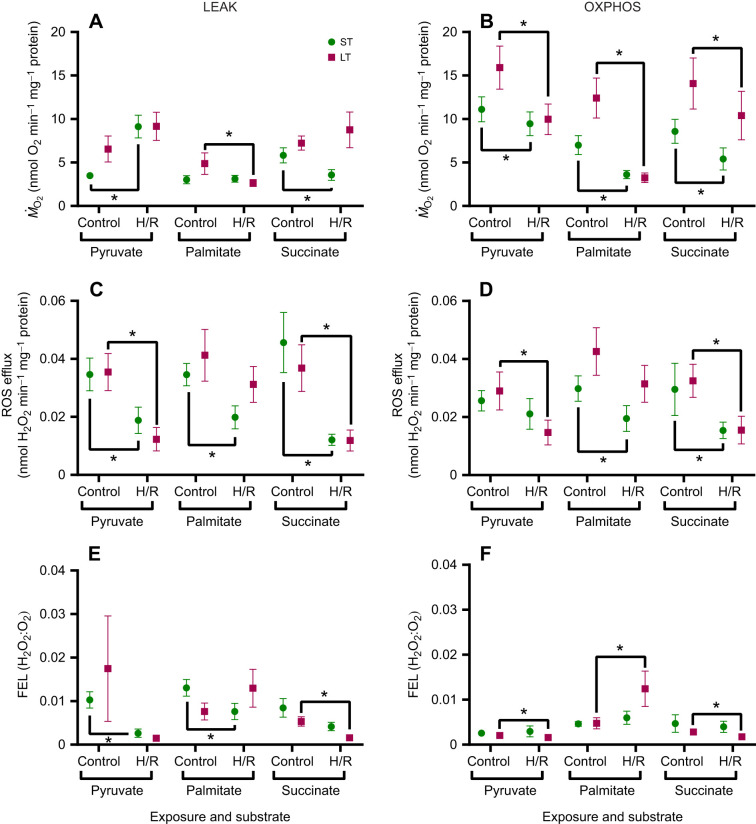
**Effects of short-term (ST) and long-term (LT) H/R on LEAK and OXPHOS respiration of *P. maximus* digestive gland mitochondria.** *Ṁ*_O_2__ (A,B), ROS efflux (C,D) and FEL (E,F) in resting (LEAK; left) and active (OXPHOS; right) mitochondria oxidizing pyruvate, palmitate and succinate. Significant effects of hypoxia exposure (between control and H/R) on a particular mitochondrial trait are represented by asterisks (*P<0.05). *N*=8 for all substrates except palmitate (*N*=7 for palmitate-driven ROS and FEL in long-term hypoxia).

Similar to the gill mitochondria, H/R resulted in increased LEAK *Ṁ*_O_2__ in the digestive gland mitochondria oxidizing pyruvate, although this effect was only statistically significant after short-term H/R (Fig. 3A). However, during palmitate and succinate oxidation, LEAK *Ṁ*_O_2__ in the digestive gland mitochondria decreased after long-term (with palmitate) and short-term (with succinate) H/R (Fig. 3A). The ROS efflux in the LEAK state decreased after H/R exposure of the digestive gland mitochondria irrespective of the oxidized substrate (Fig. 3C). FEL also decreased in the digestive gland mitochondria oxidizing NADH-linked substrate (pyruvate and palmitate) after short-term H/R, whereas for long-term H/R, FEL decreased only during the succinate-driven LEAK state (Fig. 3E).

### Effect of H/R stress on scallop mitochondria: OXPHOS state

Long-term H/R exposure, but not short-term H/R exposure, showed a significant interaction with oxidized substrate on OXPHOS respiration. However, no significant interactive effects were observed between H/R exposure and oxidized substrate on ROS efflux (Tables S2 and S3).

The oxygen consumption of the scallop's gill mitochondria during OXPHOS was strongly suppressed by H/R, regardless of the oxidized substrate or hypoxia duration (Fig. 2B). The ROS efflux and FEL in the OXPHOS state generally remained at baseline levels after H/R exposure, except for the increase in FEL in the palmitate-respiring mitochondria in the OXPHOS state (Fig. 2D,F).

Similar to the gills, the OXPHOS respiration in the digestive gland mitochondria of scallops was consistently suppressed by H/R, regardless of the oxidized substrate or the duration of hypoxia (Fig. 3B). Additionally, there was also a tendency for a H/R-induced decrease in ROS efflux of the digestive gland mitochondria during OXPHOS respiration, albeit the pattern was less consistent than for *Ṁ*_O_2__ (Fig. 3D). Short-term H/R had no significant effect on OXPHOS FEL, whereas the effects of long-term H/R on OXPHOS FEL were substrate dependent, showing a modest decrease for pyruvate and succinate and a strong increase for palmitate oxidation (Fig. 3F).

### Mitochondrial respiratory coupling

**Fig. 4.**
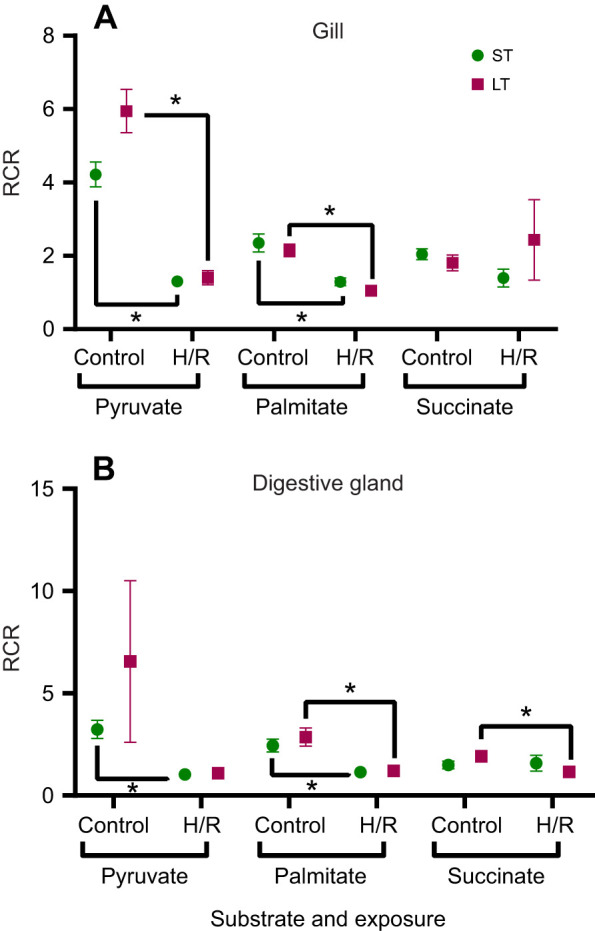
**Effects of short-term (ST) and long-term (LT) H/R stress on respiratory control ratio (RCR) of *P. maximus* gill and digestive gland mitochondria. RCR (**the ratio of OXPHOS to LEAK respiration) is shown for gill (A) and digestive gland (B) mitochondria oxidizing pyruvate, palmitate and succinate. Significant effects of hypoxia exposure (between control and H/R) on a particular mitochondrial trait are represented by asterisks (*P<0.05). *N*=8 for all substrates.

In gill and digestive gland mitochondria, exposure to both short- and long-term hypoxia suppressed mitochondrial RCR during Complex I-linked (pyruvate and palmitate) substrate oxidation (Fig. 4A,B). However, RCR remained at baseline levels during succinate-driven respiration in the gill mitochondria under both short- and long-term hypoxia conditions (Fig. 4A). In the digestive gland mitochondria, respiratory coupling with succinate decreased after long-term H/R but remained stable after short-term H/R (Fig. 4B).

### Oxidative damage after short-term hypoxia

**Fig. 5.**
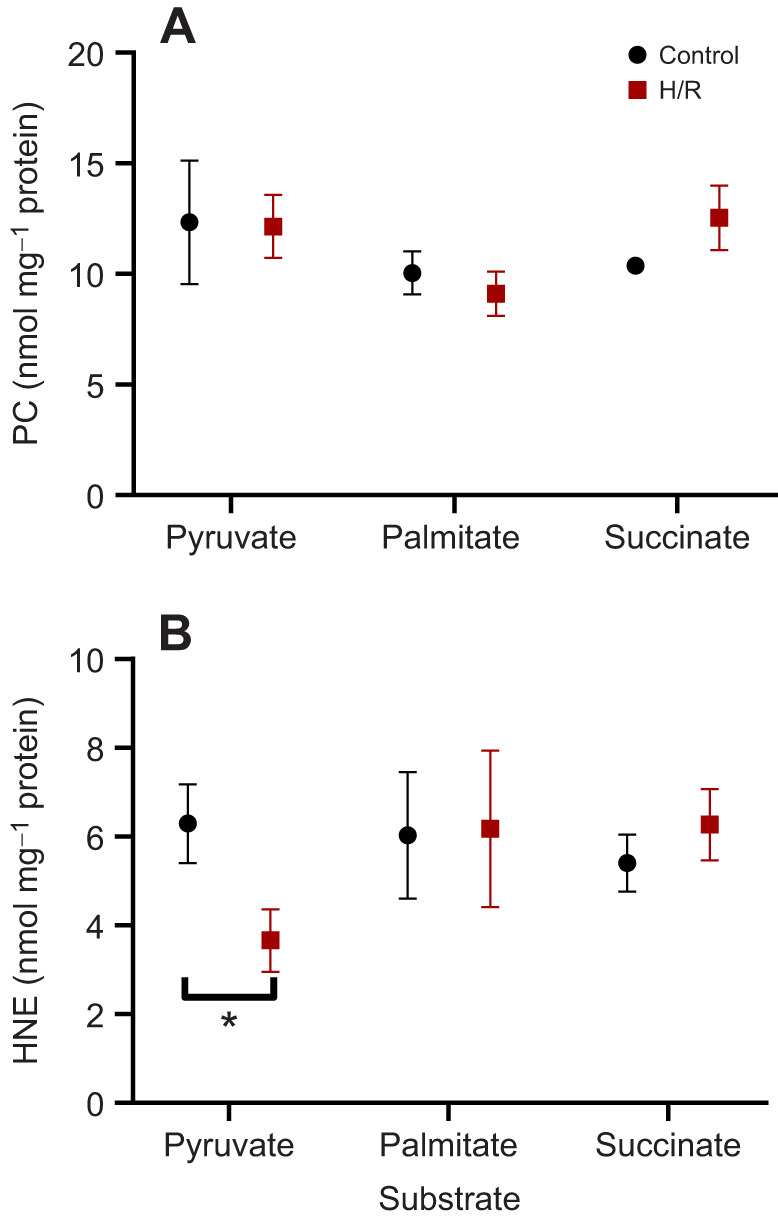
**Effects of short-term (ST) H/R stress on protein carbonyl (PC) and 4-hydroxynonenal (HNE) content of *P. maximus* gill mitochondria.** PC (A) and HNE (B) levels are shown for mitochondria oxidizing pyruvate, palmitate and succinate. Significant effects of hypoxia exposure (between control and H/R) on a particular mitochondrial trait are represented by asterisks (*P<0.05). *N*=5 for all substrates.

The analyzed oxidative stress markers showed no significant interactive or individual effects of substrate and H/R exposure (Table S4). Mitochondrial concentrations of PC and HNE generally remained at baseline levels after short-term H/R exposure, except for a significant decline in HNE levels in pyruvate-oxidizing mitochondria after H/R (Fig. 5).

## DISCUSSION

The ability of mitochondria to utilize various substrates is essential for maintaining cellular function under different physiological and metabolic conditions (Smith et al., 2018). Substrate preference varies across tissues in animals. For instance, in mammals, brain mitochondria primarily utilize glucose (Dienel and Cruz, 2015), heart mitochondria oxidize fatty acids, amino acids and lactate (Kodde et al., 2007; Lopaschuk and Stanley, 2006), liver mitochondria favor succinate (Gusdon et al., 2015), and kidney and skeletal muscle mitochondria efficiently oxidize both fatty acids and glucose (Brooks et al., 1999; Spencer and Stanton, 2017). In our study, mitochondria from the king scallop, *P. maximus*, showed a preference for pyruvate oxidation in both the gill and digestive gland tissues, as indicated by high OXPHOS rates and RCR and low ROS efflux. In contrast, mitochondria from the hypoxia-tolerant Pacific oyster *Crassostrea gigas* favored glutamate and succinate oxidation in the digestive gland, while the gill mitochondria demonstrated broader substrate oxidation preference (Adzigbli et al., 2022a). The digestive gland mitochondria in scallops generally showed higher ROS efflux and elevated electron leak rates during respiration with all three tested substrates compared with the gill mitochondria. This suggests that digestive gland tissues may be more prone to oxidative stress than gill tissues under normal oxygen conditions. The mechanisms underlying these differences, such as variations in mitochondrial antioxidant levels or the activity of alternative oxidase between tissues (Vanlerberghe, 2013), warrant further investigation.

Interestingly, both scallop (this study) and oyster (Adzigbli et al., 2022a) mitochondria showed higher ROS efflux during palmitate and succinate oxidation compared with pyruvate. Increased ROS production during palmitate oxidation has also been observed in various mammalian tissues, where it is linked to endoplasmic reticulum stress and Ca^2+^ overload, leading to oxidative stress and potential cellular damage (Dai Ly et al., 2017). Mitochondrial succinate oxidation can stimulate ROS generation via reverse electron transport (RET) through mitochondrial Complex I (Scialò et al., 2017). While RET is negligible in oyster mitochondria (Adzigbli et al., 2022a), its activity in scallop mitochondria and its possible contribution to ROS efflux remain unknown and require further investigation. In contrast, pyruvate oxidation produces NADH through pyruvate dehydrogenase, and NADH acts as an antioxidant by directly scavenging ROS and indirectly regenerating glutathione, which may reduce ROS efflux (Kirsch and Groot, 2001). This antioxidant activity of pyruvate may explain the low ROS efflux observed in scallop mitochondria when oxidizing pyruvate.

Marine bivalves of the family Pectinidae, including the king scallop, *P. maximus*, show a notable sensitivity to hypoxia when compared with other marine bivalves (Ivanina et al., 2016). Our study reveals that even relatively short periods of hypoxia (15–90 min) can reduce mitochondrial respiratory capacity, decrease mitochondrial coupling (RCR) and stimulate FEL in scallops, which is consistent with their low tolerance to hypoxia. However, the mitochondrial response to H/R stress in *P. maximus* varied depending on tissue type and substrate. The most significant decrease in ATP synthesis capacity during H/R stress occurred during palmitate oxidation (∼45–85% reduction), followed by pyruvate oxidation (∼15–55% reduction). These findings suggest that mitochondrial Complex I in *P. maximus* is highly susceptible to H/R-induced stress, which might compromise ATP synthesis from both glycolytic products and fatty acids during post-hypoxic recovery. The susceptibility of mitochondrial Complex I to H/R stress has been reported in other organisms, including turtles (Pamenter et al., 2016) and bivalves such as quahogs (Falfushynska et al., 2020), Pacific oysters and blue mussels (Adzigbli et al., 2022a; Sokolov et al., 2019). This decline in Complex I-driven OXPHOS may result from inactivation or damage to Complex I due to reversible protein phosphorylation or *S*-nitrosation of cysteine residues in Complex I (Chouchani et al., 2013; Falfushynska et al., 2020; Pamenter et al., 2016). Notably, in the Pacific oyster, *C. gigas*, H/R stress resulted in a decrease in Complex I-driven LEAK respiration, with no effect on OXPHOS (Adzigbli et al., 2022a). This suggests that Complex I in oysters can maintain high activity for OXPHOS, and the suppressed Complex I-driven LEAK respiration is probably due to factors such as modulation of proton conductance or a reduction in resting mitochondrial membrane potential. In contrast, *P. maximus* mitochondria exhibited a significant increase in pyruvate-driven LEAK respiration (∼40–165% increase, depending on tissue type and hypoxia duration), which reflects futile proton leak not associated with ATP synthesis. These results indicate that, unlike the hypoxia-tolerant oyster mitochondria, which maintain high OXPHOS and mitigate futile proton leak after H/R stress, the mitochondria of hypoxia-intolerant scallops demonstrate the opposite pattern with declining Complex I-driven OXPHOS and increasing LEAK rates.

In scallop gill and digestive gland mitochondria, Complex II-linked OXPHOS capacity declined by ∼20–40% following H/R stress. Similarly, Ivanina et al. (2016) reported a significant decline in succinate oxidation and succinate-driven OXPHOS rates in scallop (*Argopecten irradians*) mitochondria exposed to intermittent hypoxia. In contrast, mitochondria from hypoxia-tolerant bivalves maintain or even enhance OXPHOS respiration capacity when oxidizing succinate after H/R stress (Adzigbli et al., 2022a; Sokolov et al., 2019). Succinate is a key metabolic intermediate that accumulates during hypoxia-induced anaerobiosis in bivalves (Haider et al., 2020; van Hellemond et al., 2003). Upon reoxygenation, enhanced succinate oxidation facilitates post-hypoxic recovery of hypoxia-tolerant bivalves by rapidly restoring ATP levels and clearing excess succinate (Haider et al., 2020; Ouillon et al., 2021, 2023). Our study indicates that succinate-driven respiration, particularly OXPHOS, is impaired by H/R stress in scallops, in contrast to its stability or enhancement in hypoxia-tolerant species. Nevertheless, its decline was less pronounced than that of pyruvate- and palmitate-driven respiration, suggesting that scallop mitochondria might be less susceptible to H/R stress when oxidizing succinate compared with Complex I-linked substrates. This hypothesis is further supported by the ability of scallop mitochondria to maintain normal respiratory coupling (RCR) during succinate oxidation, except for a moderate decline following long-term H/R stress in the digestive gland. In contrast, Complex I-driven respiration exhibited a significant loss of RCR after both short- and long-term H/R stress. This preference for succinate as a substrate under stress conditions is consistent with findings in other invertebrate mitochondria, where succinate oxidation was shown to support a considerably higher and more robust rate of ATP synthesis under intermittent hypoxia (Adzigbli et al., 2024, 2022b) or heat stress (Jørgensen et al., 2021; Roussel et al., 2023).

Transitioning from hypoxia to high oxygen conditions often leads to increased ROS production, primarily due to the accumulation of reduced intermediates such as succinate and reduced co-enzyme Q in mammalian mitochondria (Michiels, 2004). In vertebrates, succinate is a key driver of RET through mitochondrial Complex I, leading to elevated superoxide generation (Andrienko et al., 2017; Robb et al., 2018; Wijermars et al., 2016). To mitigate RET-induced ROS production, vertebrates typically suppress Complex I activity and/or regulate succinate accumulation (Bundgaard et al., 2019, 2023; Prag et al., 2023). In bivalves, succinate accumulates as a major end product of anaerobic metabolism during hypoxia (Bayne, 2017; Haider et al., 2020; Kurochkin et al., 2009). However, in scallops, amino acid derivatives (opines) also accumulate under hypoxia and functional anaerobiosis caused by physical activity (Enomoto et al., 2000; Harcet et al., 2013). Although the present study did not directly measure RET, our findings suggest that *P. maximus* mitochondria effectively regulate ROS efflux and electron leak during H/R stress, regardless of the oxidized substrate. In the gills, the substrate-specific ROS release rate remained unchanged in both resting and phosphorylating mitochondria after H/R stress, except for a decrease in ROS efflux during succinate oxidation in the LEAK state. Notably, FEL (the ratio of H_2_O_2_ efflux to O_2_ consumption) remained low and stable in gill mitochondria respiring on pyruvate or succinate. In contrast, FEL increased during palmitate oxidation, indicating that fatty acid oxidation under H/R stress is associated with elevated oxidative stress in scallop gills. However, despite this increase, the physiological impact appears minimal, as no oxidative damage to proteins or lipids was detected in mitochondria following H/R stress, irrespective of the oxidized substrate.

Unlike in gill mitochondria, H/R stress reduced ROS release in scallop digestive gland mitochondria by ∼15–70% during NADH-linked substrate oxidation and by ∼35–75% during succinate oxidation in both LEAK and OXPHOS states. However, this decrease was not fully proportional to the reduction in oxygen consumption rates, as evidenced by changes in FEL after H/R stress. Our results indicate that the oxidation of pyruvate and succinate benefits the digestive gland after H/R stress, as these substrates are associated with lower ROS efflux and FEL. This suggests that digestive gland mitochondria may be more efficient at ROS consumption than those of the gills, potentially as a result of higher antioxidant levels linked to their elevated baseline ROS production, and implies that oxidative stress is unlikely to be the primary cause of mitochondrial respiratory decline in the digestive gland mitochondria following H/R stress. In contrast, during palmitate oxidation, FEL was suppressed following short-term H/R stress but significantly increased after long-term H/R stress, indicating a decrease in electron transfer efficiency in the latter case. These findings support the hypothesis that fatty acid oxidation is notably dysregulated during post-hypoxic recovery in both scallop tissues. A similar dysregulation of fatty acid oxidation during post-hypoxic recovery has also been reported in Pacific oyster digestive gland mitochondria (Adzigbli et al., 2022a).

Overall, our study of mitochondrial responses of *P. maximus* to H/R stress reveals both resilience and susceptibility in its metabolic response. While scallop mitochondria exhibit a preference for pyruvate oxidation under normoxic conditions, H/R stress led to a significant decline in Complex I-driven ATP synthesis, increased proton leak and dysregulated fatty acid oxidation, particularly in the digestive gland. In contrast, succinate oxidation was less affected, suggesting a potential role in post-hypoxic recovery, as seen in other invertebrates (Adzigbli et al., 2024, 2022b; Jørgensen et al., 2021; Roussel et al., 2023). The ability to maintain succinate oxidation aligns with strategies observed in hypoxia-tolerant species (Sokolova et al., 2019), but the overall decline in mitochondrial function underscores the scallop's vulnerability to hypoxia. These findings suggest that *P. maximus* lacks the metabolic flexibility observed in more tolerant bivalves, making it susceptible to hypoxia-induced stress. Future research should investigate whether mechanisms such as reversible post-translational modifications or alternative oxidase activation contribute to species-specific differences in mitochondrial resilience.

## Supplementary Material

10.1242/jexbio.249870_sup1 Supplementary information
