# Supplementary material for: Effects of hypoxia–reoxygenation on the bioenergetics and oxidative stress in the isolated mitochondria of the king scallop, Pecten maximus
Source: J Exp Biol. 2025 May 12;228(9):jeb249870. doi: 10.1242/jeb.249870 (PMC12091870; doi:10.1242/jeb.249870)
Supplement: Supplementary information [file jexbio-228-249870-s1.pdf]

Table S1. Repeated measures two-way ANOVA: Effects of tissue type (gills and digestive gland), oxidized substrates and their interactions on the MO_2, ROS efflux and FEL in the mitochondria of *P. maximus* under normal oxygen conditions.

Mitochondrial functional indices measurement during LEAK and OXPHOS respiration. ANOVA values are presented as F-values (degrees of freedom for the effect and the error in subscript) and P-values. Significant effects (P<0.05) are highlighted in bold.

	Tissue	Substrate	Interactive effect
$\dot{M}O_2$ LEAK	$F_{1,45}$=1.181, P=0.283	**$F_{2,45}$=11.037, P<0.001**	$F_{2,45}$=0.069, P=0.933
$\dot{M}O_2$ OXPHOS	$F_{1,45}$=3.544, P=0.066	**$F_{2,45}$=20.950, P<0.001**	**$F_{2,45}$=5.193, P=0.009**
ROS LEAK	**$F_{1,42}$=24.742, P<0.001**	**$F_{2,42}$=4.480, P=0.017**	$F_{2,16}$=1.071, P=0.352
ROS OXPHOS	**$F_{1,42}$=32.789, P<0.001**	**$F_{1,42}$=3.612, P=0.036**	$F_{2,42}$=0.300, P=0.742
FEL LEAK	**$F_{1,41}$=22.173, P<0.001**	**$F_{2,41}$=4.081, P=0.024**	$F_{2,41}$=1.992, P=0.149
FEL OXPHOS	**$F_{1,42}$=21.189, P<0.001**	**$F_{2,42}$=7.592, P=0.002**	$F_{2,42}$=0.767, P=0.471

Journal of Experimental Biology • Supplementary information

Table S2. Repeated measures two-way ANOVA: Effects of short-term H-R exposure, oxidized substrates and their interactions on the MO_2, ROS efflux, and FEL in the gill and the digestive gland (DG) mitochondria of *P. maximus*.

Mitochondrial functional indices measurement during LEAK and OXPHOS respiration. ANOVA values are presented as F-values (degrees of freedom for the effect and the error in subscript) and P-values. Significant effects (P<0.05) are highlighted in bold.

	Exposure	Substrate	Interactive effect
Mo₂			
$LEAK_{GILL}$	**$F_{1,21}$=5.980, P=0.023**	**$F_{2,21}$=16.323, P<0.001**	**$F_{2,21}$=23.169, P<0.001**
$LEAK_{DG}$	**$F_{1,22}$=5.235, P=0.032**	**$F_{1,22}$=8.302, P=0.002**	**$F_{2,22}$=20.551, P<0.001**
$OXPHOS_{GILL}$	**$F_{1,21}$=113.114, P<0.001**	**$F_{2,21}$=19.827, P<0.001**	$F_{2,21}$=0.016, P=0.984
$OXPHOS_{DG}$	**$F_{1,22}$=50.012, P<0.001**	**$F_{2,22}$=4.982, P=0.016**	$F_{2,22}$=1.951, P=0.166
ROS			
$LEAK_{GILL}$	**$F_{1,20}$=11.148, P=0.003**	**$F_{2,19}$=4.617, P=0.022**	**$F_{2,20}$=15.287, P<0.001**
$LEAK_{DG}$	**$F_{1,22}$=42.340, P<0.001**	$F_{2,22}$=0.053, P=0.949	$F_{2,22}$=3.411, P=0.051
$OXPHOS_{GILL}$	$F_{1,20}$=3.686, P=0.069	**$F_{2,20}$=4.453, P=0.025**	$F_{2,20}$=0.577, P=0.571
$OXPHOS_{DG}$	**$F_{1,22}$=14.929, P=0.001**	$F_{2,22}$=0.053, P=0.948	$F_{2,22}$=1.201, P=0.320
FEL			
$LEAK_{GILL}$	$F_{1,21}$=0.704, P=0.411	$F_{2,21}$=1.914, P=0.172	$F_{2,21}$=1.774, P=0.194
$LEAK_{DG}$	**$F_{1,22}$=35.650, P<0.001**	$F_{2,22}$=2.467, P=0.108	$F_{2,22}$=0.996, P=0.385
$OXPHOS_{GILL}$	**$F_{1,20}$=6.440, P=0.020**	**$F_{2,20}$=5.271, P=0.015**	**$F_{2,20}$=4.813, P=0.020**
$OXPHOS_{DG}$	$F_{1,22}$=0.338, P=0.567	$F_{2,22}$=1.299, P=0.293	$F_{2,22}$=1.076, P=0.365

Table S3. Repeated measures two-way ANOVA: Effects of long-term H-R exposure, oxidized substrates and their interactions on the MO_2, ROS efflux, and FEL in the gill and the digestive gland (DG) mitochondria of *P. maximus*.

Mitochondrial functional indices measurement during LEAK and OXPHOS respiration. ANOVA values are presented as F-values (degrees of freedom for the effect and the error in subscript) and P-values. Significant effects (P<0.05) are highlighted in bold.

	Exposure	Substrate	Interactive effect
MO_2			
LEAK $_{GILL}$	$F_{1,21}$=0.310, P=0.584	**$F_{2,19}$=7.955, P=0.003**	**$F_{2,21}$=26.189, P<0.001**
LEAK $_{DG}$	$F_{1,20}$=0.504, P=0.486	**$F_{2,20}$=3.928, P=0.036**	$F_{2,20}$=2.576, P=0.101
OXPHOS $_{GILL}$	**$F_{1,21}$=188.101, P<0.001**	**$F_{2,21}$=30.773, P<0.001**	**$F_{2,21}$=25.472, P<0.001**
OXPHOS $_{DG}$	**$F_{1,20}$=56.209, P<0.001**	$F_{2,20}$=1.489, P=0.250	**$F_{2,20}$=3.529, P=0.049**
ROS			
LEAK $_{GILL}$	$F_{1,19}$=3.366, P=0.082	**$F_{2,19}$=8.878, P=0.002**	$F_{2,19}$=2.697, P=0.093
LEAK $_{DG}$	**$F_{1,20}$=32.385, P<0.001**	$F_{2,20}$=1.460, P=0.256	$F_{2,20}$=1.822, P=0.188
OXPHOS $_{GILL}$	$F_{1,20}$=0.021, P=0.885	**$F_{2,20}$=17.011, P<0.001**	$F_{2,20}$=0.709, P=0.504
OXPHOS $_{DG}$	**$F_{1,20}$=36.652, P<0.001**	$F_{2,20}$=2.017, P=0.159	$F_{2,20}$=0.516, P=0.604
FEL			
**LEAK $_{GILL}$**	**$F_{1,19}$=10.815, P=0.004**	**$F_{2,19}$=18.403, P<0.001**	**$F_{2,19}$=13.407, P<0.001**
**LEAK $_{DG}$**	$F_{1,21}$=1.252, P=0.276	$F_{2,21}$=0.949, P=0.403	$F_{2,21}$=2.081, P=0.150
**OXPHOS $_{GILL}$**	**$F_{1,20}$=77.255, P<0.001**	**$F_{2,20}$=69.665, P<0.001**	**$F_{2,20}$=66.748, P<0.001**
**OXPHOS $_{DG}$**	**$F_{1,20}$=5.472, P=0.030**	**$F_{2,20}$=6.973, P=0.005**	**$F_{2,20}$=9.871, P=0.001**

Journal of Experimental Biology • Supplementary information

Table S4. Repeated measures two-way ANOVA: Effects of short-term H-R exposure, oxidized substrates and their interactions on the oxidative stress indices of gill mitochondria of *P. maximus*

	Exposure	Substrate	Interactive effect
Carbonyls	$F_{1,11}$=0.303, P=0.593	$F_{1,11}$=2.076, P=0.172	$F_{2,11}$=0.717, P=0.509
HNE	$F_{1,12}$=0.288, P=0.601	$F_{1,12}$=0.714, P=0.510	$F_{2,12}$=1.125, P=0.357
